# Qualitative and quantitative methods show stability in patterns of *Cepaea nemoralis* shell polymorphism in the Pyrenees over five decades

**DOI:** 10.1002/ece3.7443

**Published:** 2021-03-23

**Authors:** Daniel Ramos‐Gonzalez, Angus Davison

**Affiliations:** ^1^ School of Life Sciences University of Nottingham Nottingham UK

## Abstract

Over the past century, the study of animal color has been critical in establishing some of the founding principles of biology, especially in genetics and evolution. In this regard, one of the emerging strengths of working with the land snail genus *Cepaea* is that historical collections can be compared against modern‐day samples, for instance, to understand the impact of changing climate and habitat upon shell morph frequencies. However, one potential limitation is that prior studies scored shell ground color by eye into three discrete colours yellow, pink, or brown. This incurs both potential error and bias in comparative surveys. In this study, we therefore aimed to use a quantitative method to score shell color and evaluated it by comparing patterns of *C. nemoralis* shell color polymorphism in the Pyrenees, using both methods on present‐day samples, and against historical data gathered in the 1960s using the traditional method. The main finding was that while quantitative measures of shell color reduced the possibility of error and standardized the procedure, the same altitudinal trends were recovered, irrespective of the method. The results also showed that there was a general stability in the local shell patterns over five decades, including altitudinal clines, with just some exceptions. Therefore, although subject to potential error human scoring of snail color data remains valuable, especially if persons have appropriate training. In comparison, while there are benefits in taking quantitative measures of color in the laboratory, there are also several practical disadvantages, mainly in terms of throughput and accessibility. In the future, we anticipate that genomic methods may be used to understand the potential role of selection in maintaining shell morph clines. In addition, photographs generated by citizen scientists conducting field surveys may be used with deep learning‐based methods to survey color patterns.

## Graphical abstract

We used a quantitative method to score the shell colour of *Cepaea nemoralis* shells, and evaluated it by comparing patterns shell colour polymorphism in the Pyrenees, using both methods on present day samples, and against historical data gathered in the 1960s using the traditional method. The main finding was that while quantitative measures of shell colour reduced the possibility of error and standardised the procedure, the same altitudinal trends were recovered, irrespective of the method. The results also showed that there was a general stability in the local shell patterns over five decades, including altitudinal clines, with just some exceptions.

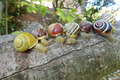

## 1 INTRODUCTION

Over the past century, the study of animal color has been critical in establishing some of the founding principles of biology, especially in genetics and evolution. More specifically, inherited variation in animal color has been used to understand the relative roles that natural selection and random genetic drift have to play in the establishment and maintenance of color polymorphism. In this respect, two of the most important species in studying color polymorphism—alongside the peppered moth (Cook, 2003)—have been the west European land snails *Cepaea nemoralis* and *C. hortensis*. This is partly because individuals are relatively easy to collect and study, the color and banding morphs show Mendelian inheritance (Cain & Sheppard, 1950, 1952, 1954; Jones et al., 1977; Lamotte, 1959), but also because one of the continuing benefits of working with *Cepaea* is an ability to compare the changes in the relative frequencies of shell color morphs over several decades. Of particular use, the “Evolution Megalab” project digitized a large set of 20th century samples (Cameron & Cook, 2012; Silvertown et al., 2011; Worthington et al., 2012). These records, and others deposited in museums, are now being used with modern surveys to produce an increasing number of comparative papers (Cameron & Cook, 2012; Cameron et al., 2013; Cook, 2014; Cowie & Jones, 1998; Ożgo & Schilthuizen, 2012; Silvertown et al., 2011; Worthington et al., 2012).

In nearly all comparative studies of *Cepaea* reported to date, absolute change in frequencies of the main shell morphs, color, and banding has been reported, but the direction is not always consistent. The conclusions are in part dependent upon the geographic scale and the precision of resampling, whether exact or nearest neighbor. To fully understand changes (or stasis) in shell polymorphism, both global and local surveys are needed (Berjano et al., 2015). For instance, large‐scale surveys illustrate the broad picture of the changes in the spatial variation of the polymorphism. In the largest study, a historical dataset of more than six thousand population samples of *C. nemoralis* was compared with new data on nearly three thousand populations (Silvertown et al., 2011). A historic geographic cline among habitats in the frequency of the yellow shells was shown to have persisted into the present day. However, there was also an unexpected decrease in the frequency of unbanded shells, and a corresponding increase in frequency of banded and midbanded morph particularly (Silvertown et al., 2011). A UK‐wide study also used Evolution Megalab data, but reported a somewhat different pattern of change. Yellow and midbanded morphs had increased in woodland, whereas unbanded and midbanded increased in hedgerow habitats (Cook, 2014).

In comparison with these large surveys, the majority of comparative studies have been at a more local scale. The benefit of these is that resampling is often precise (Cameron et al., 2013; Cook et al., 1999; Cowie & Jones, 1998; Ożgo et al., 2017; Ożgo & Schilthuizen, 2012), and it is also possible to take local factors into account. Most of the original historic studies took place in the UK. Following resampling, modern comparative surveys have tended to find an increase in yellow and midbanded shells (as above) (Cameron et al., 2013; Ożgo et al., 2017; Ożgo & Schilthuizen, 2012; Silvertown et al., 2011), but with exceptions (Cameron & Cook, 2012; Cook et al., 1999; Cowie & Jones, 1998), depending upon the precise scale of comparison. Moreover, patterns of change are not always consistent within the same study.

One potential limitation of all of these works is that shell ground color was scored by eye, usually in the field, into three discrete colors yellow, pink, or brown. Even if persons are trained, there is still bias and error, and potential for dispute over what defines each color. In practise, it is frequently difficult to distinguish the colors, and define different shades of the same color. Therefore, to understand whether color variation is in reality continuous, and to investigate how the variation may be perceived by an avian predator, psychophysical models of color vision were applied to shell reflectance measures, finding that both achromatic and chromatic variation are continuously distributed over many perceptual units in indiscrete in *Cepaea nemoralis* (Davison et al., 2019). Nonetheless, clustering analysis based on the density of the distribution did reveal three groups, roughly corresponding to human‐perceived yellow, pink, and brown shells.

**FIGURE 1:**
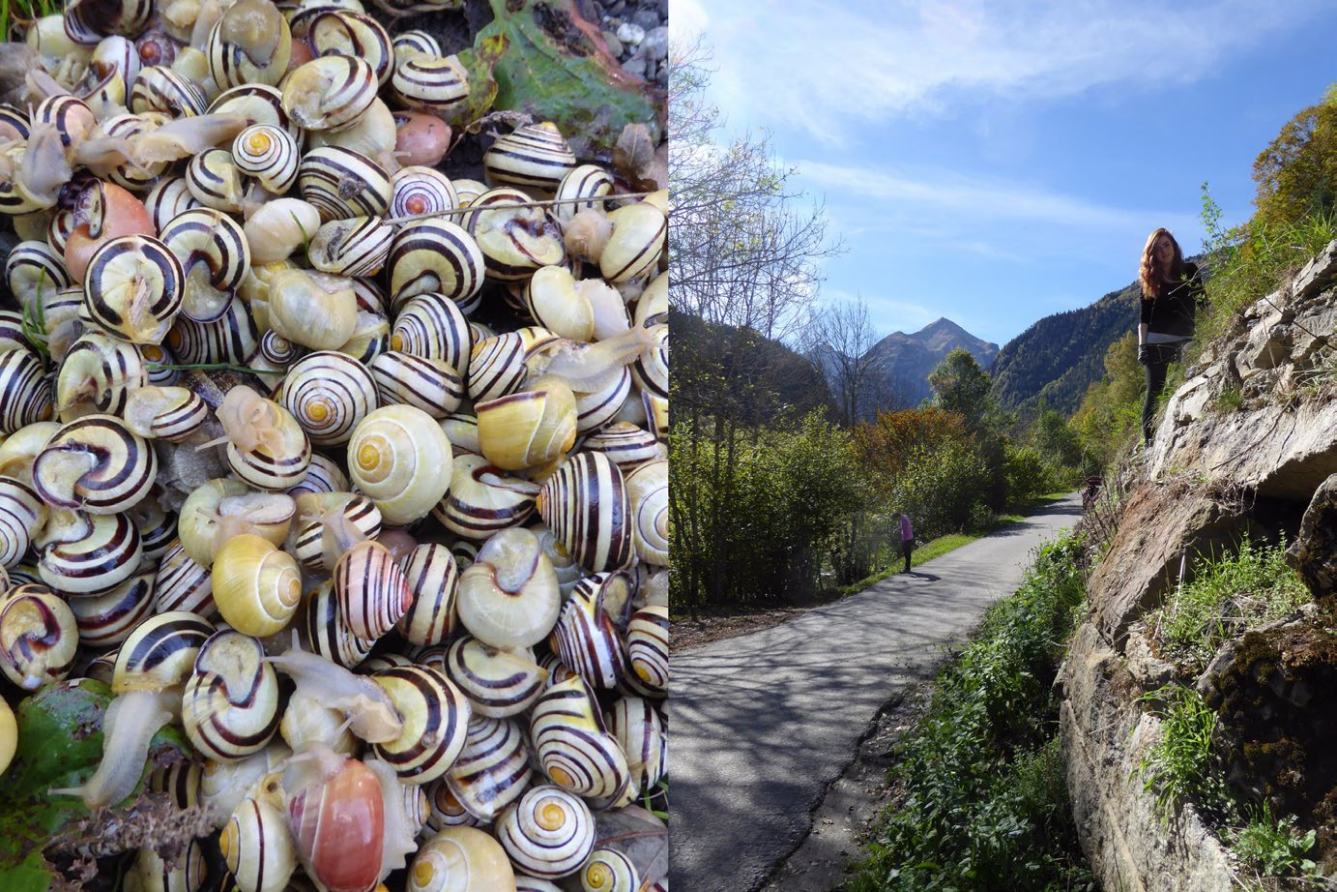
Left: variety of *Cepaea nemoralis* color morphs found in the Pyrenees. Right: typical habitat in which they are found

**FIGURE 2:**
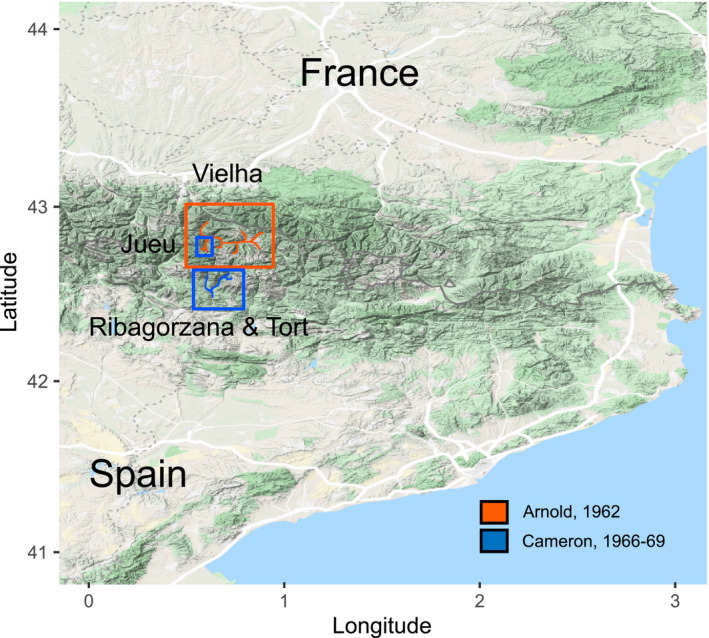
Overview of sampling locations in the Pyrenees, including this work, and previous work by others in the 1960s (Arnold, 1968; Cameron et al., 1973)

This prior work raised the possibility that reproducible, quantitative shell color measures, based on spectrophotometry in the laboratory, can be used to compare and test regular shell color data, avoiding the requirement to bin measures into color categories. In this study, we therefore aimed (1) to use the quantitative method to score shell color, and (2) evaluated it by comparing patterns of *C. nemoralis* shell color polymorphism using both methods on present‐day samples, and against historical data gathered using the traditional method. To achieve this aim, the Central Pyrenees were used as an exemplar location, because they were intensively surveyed during the 1960s and 70s (Figures 1, 2 and 1, 2), sometimes showing sharp discontinuities of frequencies of morphs within and between valleys.

The main finding was that while quantitative measures of shell color reduced the possibility of error, and standardized the procedure, the same altitudinal trends were recovered, irrespective of the method. There was remarkable a stability in the local shell patterns over five decades. Overall, while there are key benefits in taking quantitative measures of color in the laboratory, there are also several practical disadvantages. In the future, with the increasing use of digital cameras to capture and record species presence, there is the potential that color and banding data may be extracted from the images uploaded to public databases and apps such as iRecord, iNaturalist, and SnailSnap (Harvey, 2018; Horn et al., 2018; Kerstes et al., 2019). For the moment, the fact remains that human‐scoring of snail color data is valuable, especially with appropriate training.

## 2 MATERIALS AND METHODS

### 2.1 Shell samples and human‐scoring of shell phenotype

The Valle de Vielha, Valle de Jueu, Valle Noguera de Tort, and Valle Noguera Ribagorzana, hereafter abbreviated as “Vielha”, “Jueu”, “Tort,” and “Riba,” were selected for sampling (Figure 2). This is because they had been previously sampled in 1962 by Arnold (1968), and in 1966 and 1969 by Cameron et al. (1973), with the color and banding data made available via the Evolution Megalab database. New samples were collected in October 2017 and June 2018. By choice, we aimed to sample in the same location as described in past surveys, using the coordinates recorded in the Megalab database; when this was not possible, samples were collected from the nearest adjacent site with suitable habitat for snails.

Snail shell color was qualitatively scored in the laboratory as either yellow, pink, or brown, by DRG. Similarly, following previous convention, shells were scored as “unbanded” (00000), “midbanded” (00,300), or “banded” (all banding versions except midbanded). These three categories were used in all subsequent analyses. As *C. nemoralis* in the Pyrenees is polymorphic for other characters, we also scored the lip color, as either pale (usually white) or any other color (usually black or dark brown), and measured the shell height (H) and width (W) using a Vernier calliper with 0.05 mm precision, then calculating the shape as H/W.

### 2.2 Quantification of shell color

The ground color of adult snail shells from Vielha and Jueu valley was measured using an Ocean Optics spectrometer (model USB2000 + UV‐VIS‐ES) and a Xenon light source (DT‐MINI‐2‐GS UV‐VIS‐NIR), as described previously (Davison et al., 2019). Briefly, the shell underside was used because it is generally unbanded and the least damaged/exposed to sunlight, holding the probe at a 45° incident angle, ~2 mm from the shell. Each sample was quantified three times, nonconsecutively, recalibrating using light (WS‐1) and dark standards after 2 to 5 quantifications, software was recalibrated by using light standards (Davison et al., 2019). Data were collected using Ocean Optics SpectraSuite 2.0.162, using an integration time of 750 msec, boscar width of 5, and scans to average 10. Reflectance spectra were analyzed following a modified protocol described below (Davison et al., 2019; Delhey et al., 2015), using Pavo 2.2.0 R package to bin raw reflectance spectra (1 nm) (Maia et al., 2013, 2018), and then R version 3.4.1 (R Core Team, 2020) for further analyses (Delhey et al., 2015).

In a previous analysis, the aim was to understand how an avian predator might perceive the shell colors, so the tetrachromatic colorimetric standards of a blackbird (*Turdus merula*) were used (Davison et al., 2019). In this new analysis, the main aim was to compare human qualitative scores of shell color against quantitative scores, so as to better understand any biases. Reflectance spectra analysis was therefore analyzed using human CIE color trichromatic coordinates (Smith & Guild, 1931; Westland et al., 2012), as follows.

CIE standards are based on the stimulation of the different photoreceptors’ cells (cones) of the retina. In humans, three main groups of cones are found, L (long wavelength, peaking at 560 nm), *M* (medium wavelength, peaking at 530 nm), and S (short wavelength, peaking at 420 nm) (Hunt, 2004). The visual color spectra (300–700 nm) were converted using the three chromatic coordinates of the visual space, xyz, where Euclidean distances between points reflect perceptual differences, generated from quantum catches for each photoreceptor (Cassey et al., 2008). The human trichromatic coordinates (xyz), determined from the tristimulus values (XYZ), were calculated by Pavo 2.2.0 R package, a color spectral and spatial perceptual analysis, organization and visualization package, and the “standard daylight” (d65) irradiance spectrum (Maia et al., 2018; Smith & Guild, 1931). Then, a principal component analysis (PCA) was undertaken as described previously (Davison et al., 2019; Delhey et al., 2015; Scrucca et al., 2016).

### 2.3 Analysis of phenotype frequencies and correlation

To compare past and present‐day datasets, the change in the frequencies of color and banding traits for each sample site was calculated. To detect any overall trends in each valley, any differences were evaluated using independent paired T‐student (parametric) or paired rank Wilcoxon Test (nonparametric), selected according to normality (Shapiro–Wilk normality test) and homogeneity (*F*‐test).

Linear mixed regression models were conducted for color and banding from past and present datasets. Outliers were removed following the interquartile range method, using a Shapiro–Wilk normality test to test for deviations from normality. The Pearson correlation (parametric) or Kendall rank correlation test (nonparametric) was performed to evaluate correlation and any significance with altitude. Kendall rank correlation coefficient “Tau” were transformed into Pearson “r” coefficient to evaluate correlation and to conduct Fishers’ Z‐transformation (Fisher, 1921; Walker, 2003). The correlation breached the assumption of normality required in the standard comparative test. Therefore, Fishers’ Z‐transformation was applied to calculate the significance of the difference between the past and current correlation coefficients against altitude.

Maps, plots, and statistical tests were made using R version 3.4.1 (2017–06–30), the ggplot2 3.2.1 package for data visualization, and the ggmap 3.0.0 R package, to generate maps. Maps were acquired from the Geo‐location APIs platform in the Google maps source (https://console.cloud.google.com/apis/dashboard).

## 3 RESULTS

### 3.1 Past and present‐day geographic distribution of color and banding morphs

Snails were mainly found in open areas such as hedgerows, scrubs, meadows and grass, and rare in woodlands. In high altitude areas, snails were discovered mostly on meadows or screes. In total, snails were collected from 138 sample sites ranging from 823 m to 1921 m above sea level. However, only 108 sites and 2,633 individuals were used for the analysis, as we only considered sites with ten or more individuals collected (Table 1). Of the filtered 108 sites, 87 were judged to be the same as a previous study, based on previous coordinates, or up to 50 m distance away. In comparison, in the previous surveys, Arnold (in 1962) collected 5,006 snails from 123 sites in the Vielha and Jueu valleys (Arnold, 1968). Cameron (in 1966 and 1969) sampled 2,177 and 2,145 snails from 48 and 55 sites located in Jueu, Ribagorzana, and Tort, respectively (Cameron et al., 1973). Therefore, a total of 226 historical sample sites and 9,328 individuals were available for comparison (Table 1). Full details of all sample sites are in the Supplementary Files (Tables S1, S2, S3).

**TABLE 1:** Sampling summary. Number of sites and snails for each valley

Valley	Past (1962/1969)		Present (2017/2018)		Spectrophotometry	
	Sample sites	No. of shells	Sample sites	No. of shells	Sample sites	No. of shells
Vielha	119	4756	43	942	43	607
Jueu	49	1862	17	637	12	206
Riba	34	1545	21	518		
Tort	24	1165	27	536		
Total	226	9328	108	2633	55	813

**FIGURE 3:**
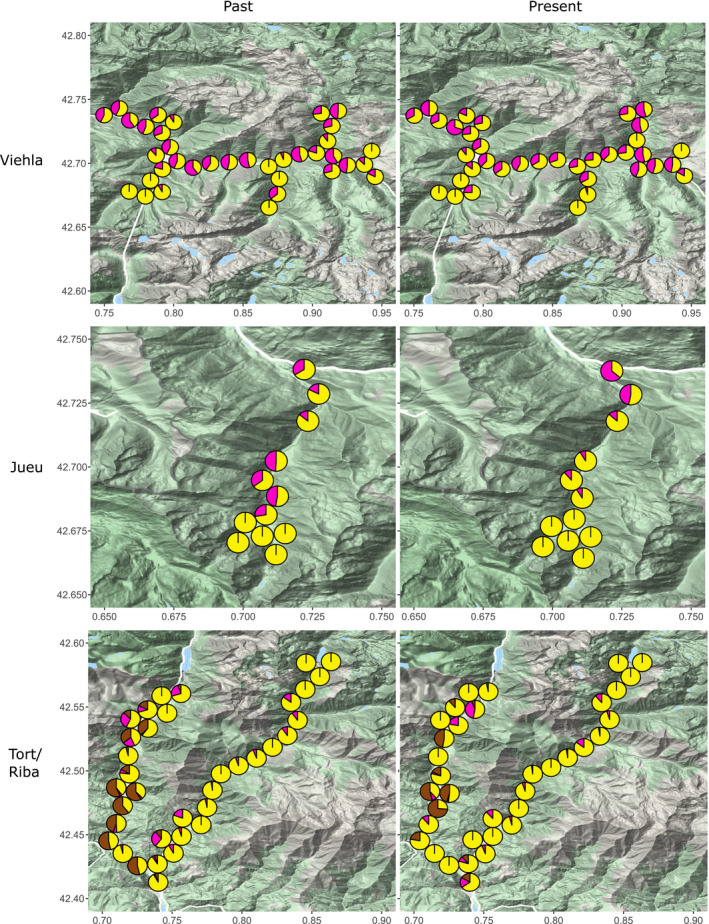
Past and present distribution of yellow, pink, and brown shell morphs in Pyrenean valleys, based on sampling in the 1960s and 2017/18. Pie charts show frequencies of yellow (yellow), pink (pink), and brown (brown) morphs in each location. Valle Noguera de Tort is the left valley, and Valle Noguera Ribagorzana is the right valley

**FIGURE 4:**
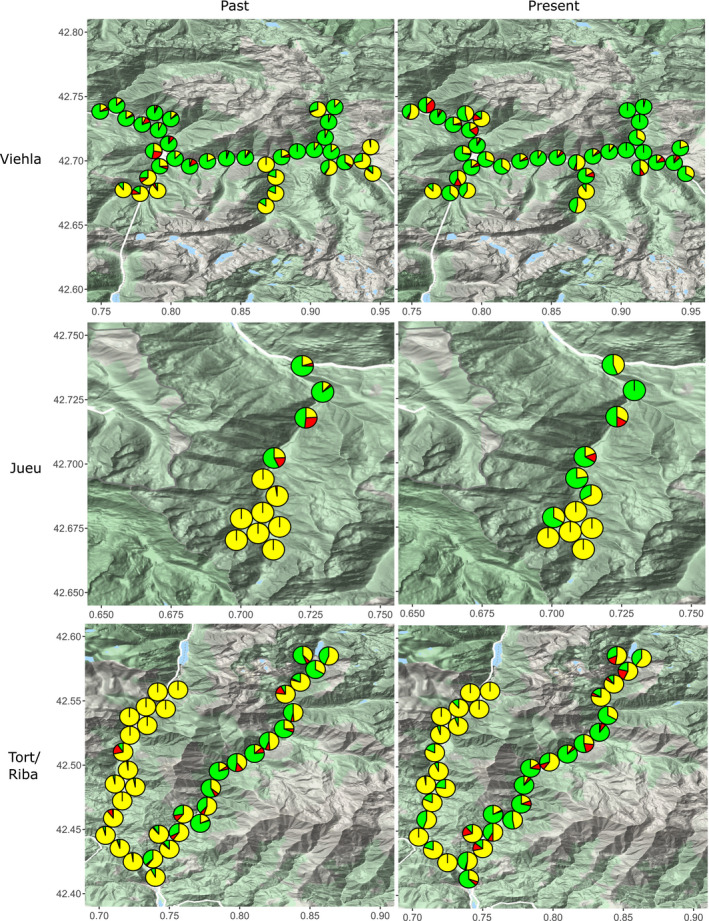
Past and present distribution of banded, midbanded, and unbanded shell morphs in four Pyrenean valleys, based on sampling in the 1960s and 2017/18. Pie charts show frequencies of banded (green), midbanded (red), and unbanded (yellow) morphs in each location. Valle Noguera de Tort is the left valley, and Valle Noguera Ribagorzana is the right valley

As in previous studies from the Pyrenees, the new survey showed that the pattern of shell morph distribution depends upon the specific valley, frequently showing associations with altitude (Figures 3 and 4). Yellow and unbanded shells tended to predominate in the higher regions of the Vielha and Jueu valleys. In the intermediate or lower sites (below ~ 1200m), pink and yellow shells had similar frequencies, with most shells also having bands. In Ribagorzana, yellow shells were commonly distributed in all sites, whereas pinks were usually found in the upper valley and brown morphs in the intermediate and lower valley. Brown populations were only found in the Ribagorzana and Tort valleys. In addition, unbanded morphs prevailed in Ribagorzana. In contrast, in the adjoining Tort valley, yellow predominated in all sites, with banded morphs predominant in almost the entire valley.

**FIGURE 5:**
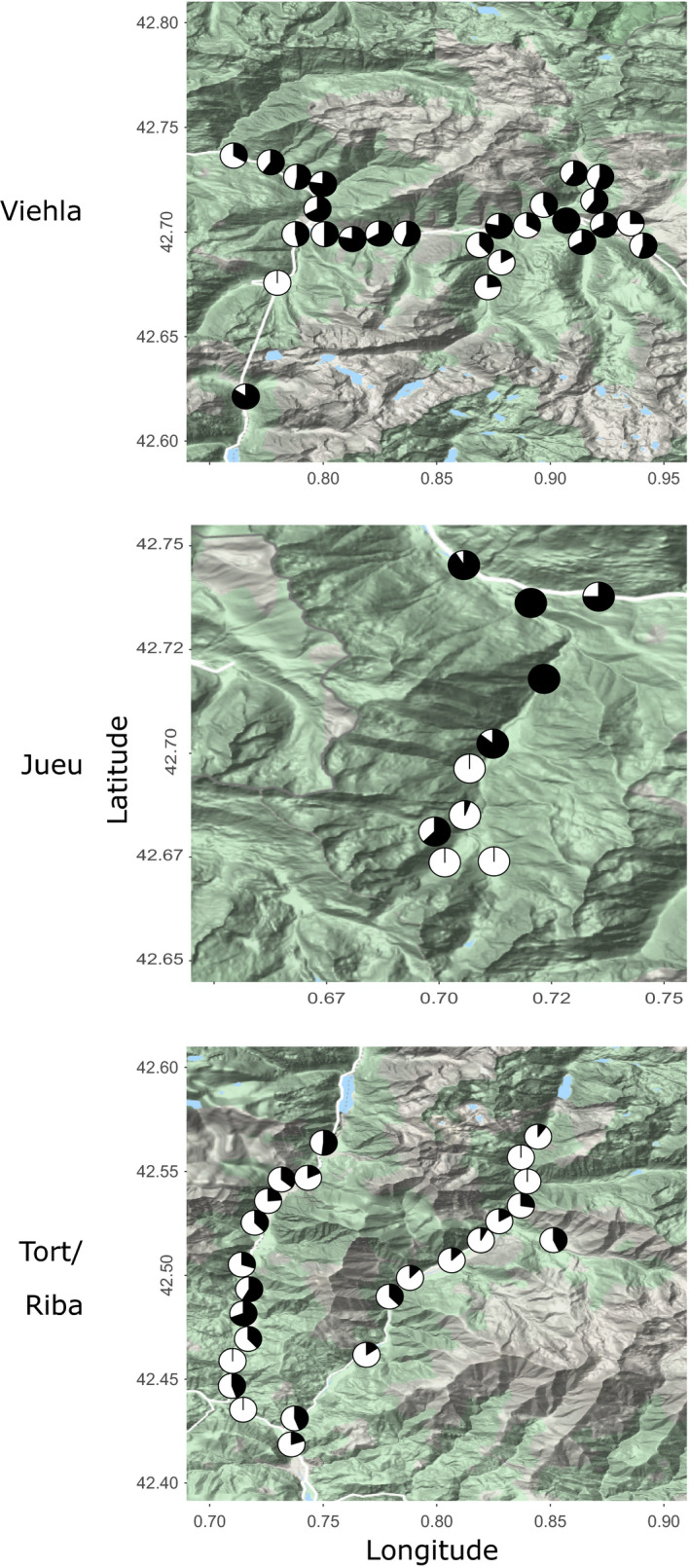
Present‐day distribution of pale‐lipped shell morphs in four Pyrenean valleys. Pie charts show frequencies of pale‐lipped shells (white) versus other forms

**FIGURE 6:**
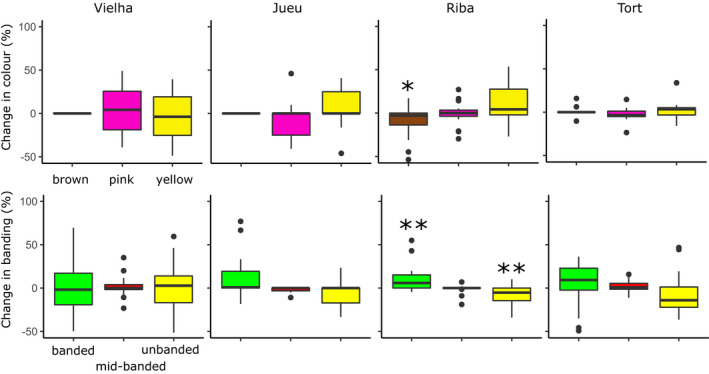
Changes in frequency of color and banding types between paired sites (same location, or within 50 m) in four Pyrenean valleys over five decades, tested using paired *t* test or Wilcoxon signed‐rank test. Ribagorzana is the only valley that showed significant changes, with the frequency of brown (*p* < 0.05) and unbanded (*p* < 0.01) shells decreasing, and the proportion of banded shells increasing (*p* <0.01)

Spatial patterns of variation in morph frequencies were largely the same as recorded in the past, including color and banding (Figures 3 and 4) as well as lip color (Figure 5). To formally test this, directional changes in the frequencies of shell types at each location between the 1960s and the present day were tested using independent paired Student's *t* test or paired rank Wilcoxon test (Tables 2, 3 and 3). This confirmed little overall change in the distribution of the main color and banding types in Vielha, Jueu, and Tort (Tables 2, 3 and 3; and Figure 6). The exception was in Ribagorzana valley, where the proportion of banded shells has risen from ~ 3% to 14%, with substantially fewer brown shells recorded and more yellow shells (Table 2).

**TABLE 2:** Statistical summary of shell geographical distribution in each valley; independent paired comparison (Student's t test (parametric), Wilcoxon signed‐rank test (nonparametric))

	Vielha				Jueu				Riba				Tort			
Present (2017/2018)	Mean	S.E.	% change	*p*‐value	Mean	S.E.	% change	*p*‐value	Mean	S.E.	% change	*p*‐value	Mean	S.E.	% change	*p*‐value
Yellow	69.4	3.4	−4.1	0.392	87.1	6.1	5.8	0.427	75.9	5.4	10.4	0.061	94.6	1.5	1.5	0.541
Pink	30.2	3.3	3.7	0.434	12.4	5.9	−6.3	0.379	9.0	3.2	−1.0	0.752	3.9	1.1	−2.3	0.204
Brown									15.4	5.2	−8.8*	0.049	1.4	1.0	0.6	0.570
Unbanded	29.7	4.1	−7.6	0.367	59.9	11.1	−13.2	0.152	85.7**	4.2	−9.6**	0.008	42.9	5.9	−6.6	0.239
Midbanded	4.9	1.3	1.8	0.192	2.6	1.8	−2.1	0.059	0.4	0.4	−1.2	0.328	8.4	1.5	2.5	0.141
Banded	66.1	4.1	6.5	0.367	37.5	10.6	15.4	0.094	13.9**	3.9	10.8**	0.007	48.7	6.3	4.1	0.499
Past (1962/1969)																
Yellow	73.5	3.4			81.3	5.6			65.5	5.7			93.0	1.4		
Pink	26.5	3.4			18.7	5.6			10.0	2.5			6.1	1.4		
Brown									24.2	5.8			0.8	0.0		
Unbanded	37.4	5.9			73.2	11.3			95.3**	1.7			49.4	5.4		
Midbanded	3.0	0.6			4.7	2.6			1.5	1.2			6.0	1.1		
Banded	59.6	5.8			22.1	9.8			3.2**	0.7			44.6	5.6		

* *p* < 0.05.

** *p* < 0.01.

*** *p* < 0.001.

**TABLE 3:** Direction of change in shell morph frequencies, from 1962/1969 to 2017/2018 in the Pyrenees

Morphs	Vielha			Jueu			Riba			Tort		
	Increase	No change	Decrease	Increase	No change	Decrease	Increase	No change	Decrease	Increase	No change	Decrease
Yellow	15	3	19	4	6	2	12	1	6	10	3	6
Pink	19	3	15	2	6	4	7	5	7	3	6	10
Brown	0	0	0	0	0	0	4	5	10	2	16	1
Unbanded	17	7	13	2	5	5	2	6	11	5	2	12
Midbanded	10	19	8	0	8	4	1	15	3	6	10	3
Banded	15	5	17	5	6	1	11	6	2	12	2	5

No Change = +/−3%.

**FIGURE 7:**
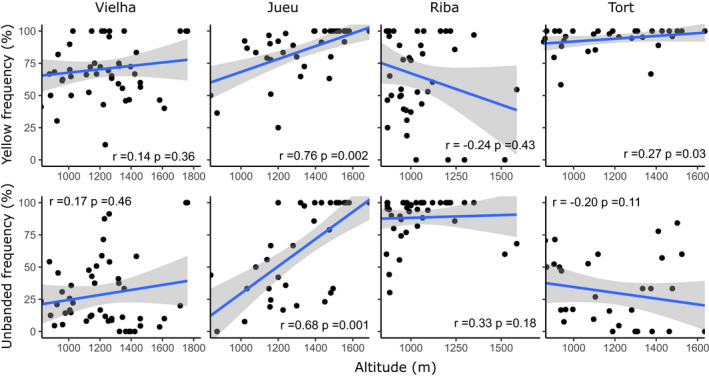
Scatterplots showing the present‐day relationship between altitude and frequency of yellow and unbanded morphs in four Pyrenean valleys. Points represent collections of shells from the same location (*n* ≥ 10). Only samples from Jueu show a significant strong positive relationship between altitude and frequency of yellow and unbanded shells; samples from Tort showed a shallow but significant association for altitude and yellow. Regression line and 95% confidence intervals are shown, alongside the Pearson coefficient and *p* value

**FIGURE 8:**
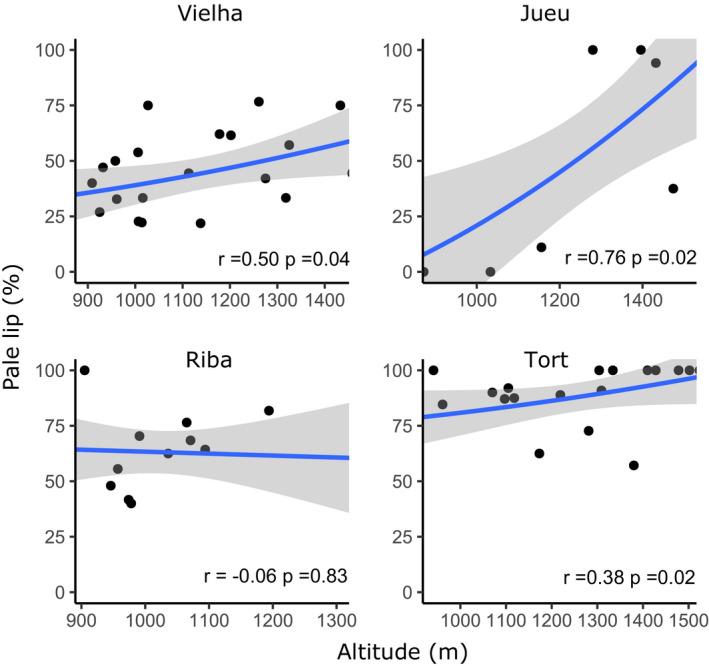
Scatterplots showing the present‐day relationship between altitude and frequency of pale‐lipped morphs in four Pyrenean valleys. Regression line and confidence intervals are shown, alongside the Pearson coefficient and *p* value

**FIGURE 9:**
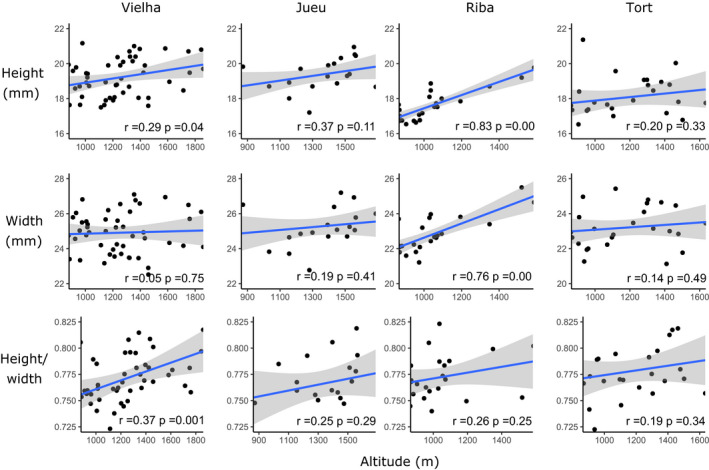
Scatterplots showing the present‐day relationship between altitude and size and shape of shells in four Pyrenean valleys. Regression line and confidence intervals are shown, alongside the Pearson coefficient and *p* value

The present‐day relationship between altitude and frequency of color and banding morphs was plotted (Figure 7). Jueu and Tort valleys showed a significant positive correlation between altitude and the frequency of yellows, with the former also showing a positive significant altitude‐unbanded association (Figure 7; Table 4). As expected, pink and banded shells showed the reverse trend, but with nonsignificant altitudinal correlations; midbanded shells did not show any correlation with altitudes. Tort showed a significant positive (but shallow) relationship between yellow‐altitude and banded‐altitude (Table 4, Figure 7, r = 0.27, 0.34, respectively, and *p* <0.05, *df* = 31). There was also significant positive association of the white‐lip morph with altitude in three valleys (Figure 8), in addition to associations of higher altitude with larger shell size (H + W), and relatively tall spires (H/W) (Figure 9).

**TABLE 4:** Statistical summary of shell altitudinal distribution in each valley, including correlations (Pearson, parametric; Kendall, nonparametric)

	Vielha			Jueu			Riba				Tort		
Present (2017/2018)	Correlation	Parametric	*p*‐value	Correlation	Parametric	*p*‐value	Correlation	Parametric	*p*‐value		Correlation	Parametric	*p*‐value
Yellow	0.136	Yes	0.357	0.755**	No	0.002	−0.235	No	0.429		0.265*	No	0.025
Unbanded	0.165	No	0.455	0.678**	No	0.001	0.326	No	0.179		−0.203	No	0.111
Banded	0.058	No	0.111	−0.687**	No	0.001	−0.070	No	0.562		0.335*	No	0.029
Spectrophotometry (2017/2018)													
Yellow	−0.081	No	0.588	0.668*	No	0.013	n/a	n/a	n/a		n/a	n/a	n/a
PC1	0.103***	No	0.000	−0.015	No	0.833	n/a	n/a	n/a		n/a	n/a	n/a
PC2	−0.088**	No	0.001	0.414***	No	0.000	n/a	n/a	n/a		n/a	n/a	n/a
PC3	−0.089	No	0.056	0.397***	No	0.000	n/a	n/a	n/a		n/a	n/a	n/a
Past (1962/1969)													
Yellow	0.482***	No	0.000	0.492***	No	0.000	−0.321	No	0.362		0.338	No	0.062
Unbanded	0.517***	No	0.000	0.858***	No	0.000	−0.059	No	0.088		−0.164	Yes	0.434
Banded	−0.480***	No	0.000	−0.834***	No	0.000	0.226	No	0.129		0.170	Yes	0.416

* *p* < 0.05.

** *p* < 0.01.

*** *p* < 0.001.

Fishers’ Z‐transformation was used to test the significance of the difference between the past and present altitudinal correlation coefficients. There were no significant changes in Jueu, Ribagorzana, and Tort (Table 5). In comparison, in the past sample from Vielha valley, both color (Table 4, yellow shells r = 0.48, *p* <0.001, *df* = 112) and banding (Table 4, unbanded shells, r = 0.51 and banded shells, r = −0.48, *p* <0.001, *df* = 112) showed a moderate association with altitude. In the present‐day, color and banding did not show a significant correlation with altitude. Thus, even though Vielha showed little overall change (Table 2), there was an increase in the proportion of pink shells at higher altitudes (e.g., increasing from 24% to 32% in sites 1,200 m and higher above sea level).

**TABLE 5:** Fisher's r‐to‐z transformation, significance of the difference between two correlation coefficients

Past vs Present	Vielha		Jueu		Riba		Tort	
	Z‐Value	*p*‐Value	Z‐Value	*p*‐Value	Z‐Value	*p*‐Value	Z‐Value	*p*‐Value
Yellow	2.120*	0.017	−1.460	0.072	−0.310	0.378	0.270	0.394
Unbanded	2.210*	0.014	1.510	0.066	−0.310	0.378	0.140	0.444
Banded	−3.16***	0.001	−1.180	0.119	1.120	0.131	−0.590	0.278
Yellow sets								
Yellow subset	3.31***	0.001	−0.740	0.230				
Yellow dataset‐subset	0.980	0.164	0.420	0.337				

* *p* < 0.05.

** *p* < 0.01.

*** *p* < 0.001

Unfortunately, it was not possible to make the same comparisons with lip color and shell measurements, because the former data were not uploaded to the Evolution Megalab database, and the size measures were not recorded in the original studies.

### 3.2 Quantitative measures of shell color and banding and associations with altitude

**FIGURE 10:**
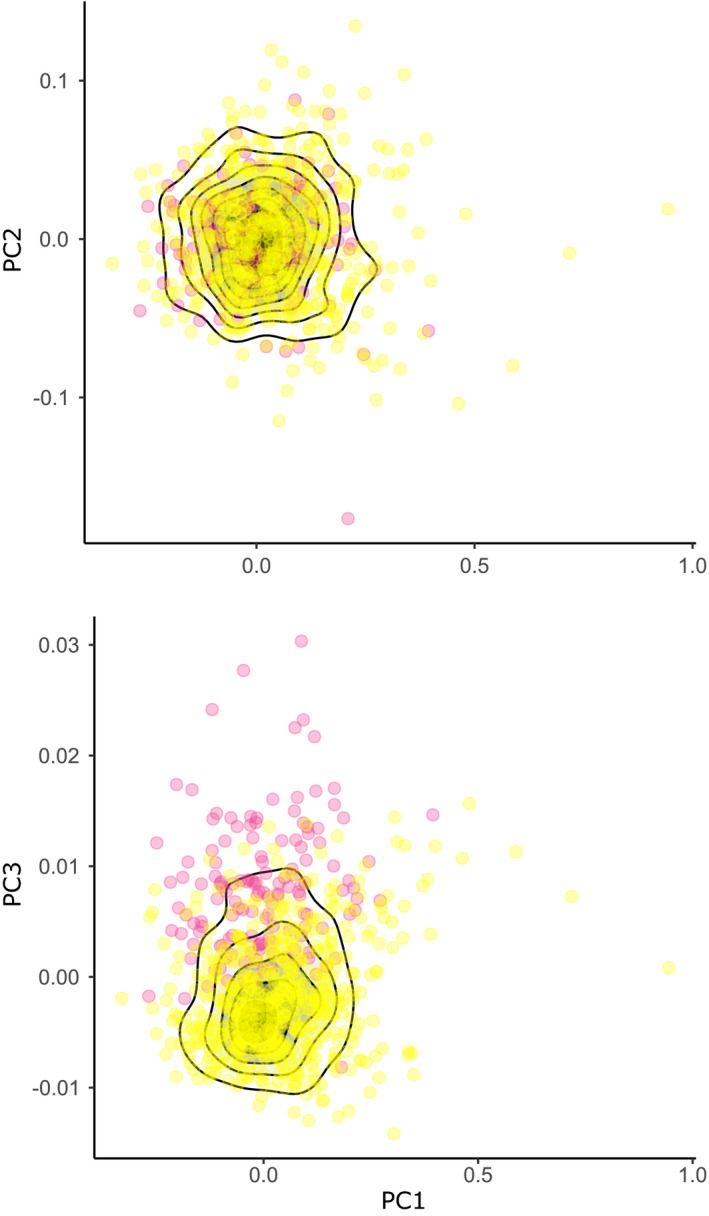
Scatterplot showing variation of visual space coordinates, xyz, on three principal component axes, using shells from Vielha and Jueu valleys in the Pyrenees. Units are in JNDs. Points are colored according to human‐scored classification of the shell, either yellow or pink

**FIGURE 11:**
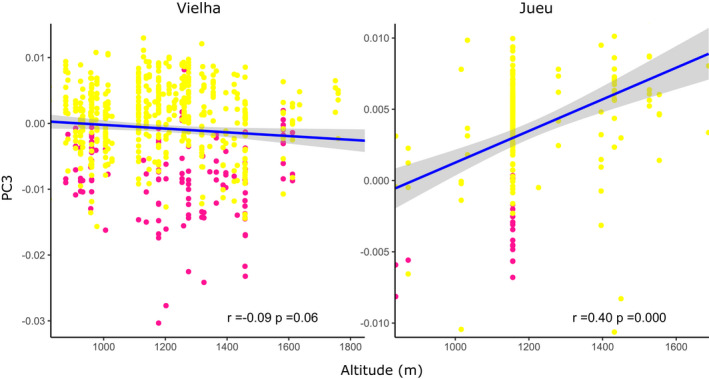
Scatterplots showing the relationship between altitude and chromatic variation (PC3) for individual shells from Vielha and Jueu valleys. Points represent individual shells, colored according to human‐scored colors. There is a strong positive association of PC3 with altitude in shells from Jueu, and a weak nonsignificant negative association in shells from Vielha. Regression line and 95% confidence intervals are shown, alongside the Pearson coefficient and *p* value

The reflectance spectra of 813 shells from Vielha and Jueu valleys were measured, a subset of the total collected (2,633; Table 1, Table S4), because some shells were too damaged to record quantitative color. A PCA on the xyz coordinates showed three axes which together explained 99% of the chromatic variation, PC1 51%, PC2 44%, and PC3 4%. As previously reported (Davison et al., 2019), the third axis, PC3, tended to separate pink and yellow shells (Figure 10). Therefore, to visualize the present‐day relationship between altitude and quantitative chromatic variation, PC3 was used because all the individuals in Vielha and Jueu were yellow or pink (Figure 11). In Vielha, there was weak negative, but nonsignificant association, of altitude and PC3, whereas Jueu showed a moderate positive correlation (Table 4). These indicate that in Vielha there was no association of shell color with altitude, whereas in Jueu yellow shells were more common at high altitude.

### 3.3 Past and present‐day associations, using qualitative and quantitative methods

We compared altitude‐color associations between historical and present‐day samples from Vielha and Jueu, using the different methods.

**FIGURE 12:**
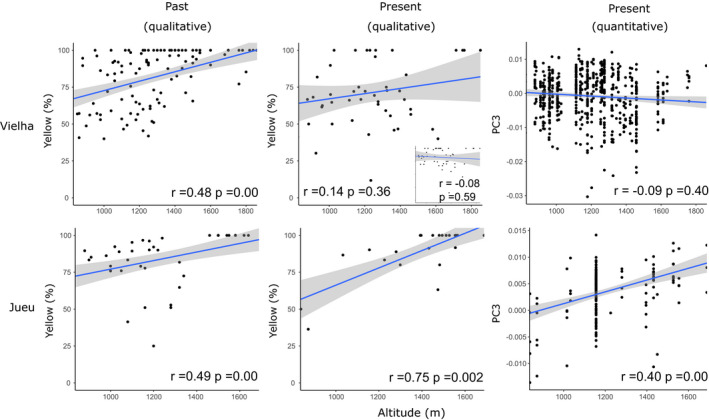
Summary figure showing the relationship between altitude and color variation for shells from Vielha and Jueu valleys, comparing past and present‐day collections, and using qualitative or quantitative methods to score color. The small inset graph shows the same data, but only using the subset of shells that were considered sufficiently undamaged for spectrophotometry

For Jueu valley (Figure 12), the same significant altitudinal associations were recovered whether using historical data (*n* = 1862), the present‐day data with human‐scoring of color (*n* = 637), or quantitative measures of color or pattern as manual scoring (*n* = 206; Figure 12). Fishers’ Z‐transformation test showed no significant changes among the altitudinal correlations for each of these four graphs (Table 5).

For Vielha valley (Figure 12), there was a significant altitudinal association with color only in the historical dataset (Table 4, *n* = 4,756, r = 0.48, *p* =0.0001, *df* = 112), compared with a non‐significant positive relationship using the present‐day data with human‐scored color (Table 4, *n* = 942, r = 0.14, *p* =0.355, *df* = 47), and a non‐significant negative relationship using quantitative measures of color (Table 4, *n* = 607, r = −0.09, *p* = 0.056, *df* = 605). To further explore these differences, we also tested for a correlation using the present‐day data with human‐scored color, but just using the subset of shells, which were considered sufficiently undamaged for spectrophotometry (Figure 12 inset graph). This showed a negative relationship (Table 4, r = −0.08, *p* = 0.588, *df* = 45), likely indicating that some (old) pink shells were mistakenly scored as yellow in the qualitative analysis.

## 4 DISCUSSION

### 4.1 Quantitative versus qualitative methods to score shell phenotype

In prior studies, the shell ground color was scored by eye, sorting individuals into three discrete categories, either yellow, pink, or brown. In this study, in addition to the human‐scoring of shell color, we evaluated a quantitative method, based on spectrophotometry in the laboratory, by comparing patterns of *C. nemoralis* shell color polymorphism from the past and the present day. The main finding was that while spectrophotometry of shell color has the benefit of being quantitative and is objective, the same trends were recovered. In fact, there was a remarkable stability in the local shell patterns in most valleys over five decades.

Both qualitative and quantitative methods have benefits and also disadvantages. Spectrophotometry produces a quantitative output for an individual shell, which better reflects the nondiscrete nature of variation in snail shell color, and is reproducible. However, it is only accessible to a few persons and requires expensive equipment, with reflectance measures are taken in the laboratory. All of these latter factors together reduce throughput. In comparison, field‐based methods do not require the snails to be taken to a laboratory, are rapid and accessible to a wide range of persons, including citizen scientists. The disadvantage is that the shell color phenotype must be binned into one of three subjective categories, with the snails from a sometimes ill‐defined single location making a single data point. Moreover, the data that are collected must be carefully filtered (e.g., Silvertown et al., 2011) to remove misidentified species (especially confusion with *C. hortensis*, juvenile *Cornu aspersum,* and *Arianta arbustorum*), a difficult task because the specimen is not preserved. Nonetheless, human‐scoring of snail color data remains valuable, especially with appropriate training.

In the future, we anticipate that a model that takes the best of both methods may be used instead. Classification by deep learning may handle a huge number of pictures and thus be more suitable for citizen science; websites and apps such as SnailSnap, iNaturalist, and iRecord (Harvey, 2018; Horn et al., 2018; Kerstes et al., 2019) are already being used extensively by the general public to capture records and images of snails, which are then identified using a combination of deep learning methods and input from persons with various degrees of expertise. For example, iNaturalist has over 9,000 observations, including photographs, of *C. nemoralis* at “research grade” quality (including > 1,000 in the UK, but only 29 in the Pyrenean region). One suggestion is that it would be relatively straightforward to extend the use of a deep learning‐based method to inspect individual images, and then record the color and the band category. Similar methods have already been implemented to identify, count, and describe the behaviors of animals in a images from motion‐sensor cameras in the Serengeti, and to recognize individual song‐birds (Ferreira et al., 2020; Norouzzadeh et al., 2018). A more sophisticated (but difficult to implement) alternative would be to also extract quantitative color data from the images, but this would have to be robust to the wide variety of circumstances under which the photographs were taken; if it was to involve some sort of color control (e.g., a card; van den Berg et al., 2020), then this would limit the number of participants.

### 4.2 Past and present‐day geographic distribution of color and banding morphs

By analyzing the geographical and altitudinal distribution of color and banding attributes in the Central Pyrenees and comparing with previous studies, we aimed to understand how local factors, human impact, and the rapid climate change acted upon the variation of *C. nemoralis* shell polymorphism. To achieve this aim, the Central Pyrenees were used as an exemplar location, because they were intensively surveyed during the 1960s and 70s, sometimes showing sharp discontinuities of frequencies of morphs (Arnold, 1968, 1969; Cameron et al., 1973; Jones & Irving, 1975) and genotypes (Ochman et al., 1983) within and between valleys. They are also particularly interesting for their geographic and ecological variation, including a diverse range of different microclimates, within and among the valleys, due to the interaction of three main climates, Atlantic, Mediterranean, and Alpine, as well as a large altitudinal differences and incidence of precipitation. Moreover, selective factors, such as climate or the human impact in the Pyrenees, have significantly changed since the 1960s (García‐Ruiz, 2015).

Broadly, we found a remarkable stability in the local shell patterns in most valleys over five decades, and associated clines, despite large changes in habitat, human impact and a rapid climate changes over five decades. Most valleys still showed similar patterns of shell types, whether color, banding, lip color or shell shape (Figures 3, 4, 5 and 9), concordant with another study over the wider Pyrenean region (Ellis, 2004). Most clines were also more or less the same (Figures 7 and 12), as has been found in other locations (Cameron et al., 2013).

There were just a few exceptions to the general pattern. For instance, the altitudinal cline in the frequency of yellows that was present in both Vielha and Jueu valleys is now only present in the latter valley. The present‐day absence of a clinal relationship is striking and contrasts with the paired comparisons at each location, which did not show any significant change in the frequency of yellow or pink in Vielha over the decades (Figure 6). The explanation for the discrepancy (Table 3) is perhaps that while pinks have increased in frequency above about 1,200 m in Vielha, we did not sample snails at the higher altitudes compared with the past, where the shells are much more likely to be yellow. Vielha is interesting because the establishment of Baqueira‐Beret ski resort (now the largest in Spain) has led to an increase of human activity and the construction of infrastructure such as dams, tunnels, or mines, with a corresponding growth of urban areas in the adjoining tributary valleys. In comparison, the Jueu valley has remained largely intact, perhaps because it is a protected reserve.

In our opinion, the most likely explanation for the loss of altitudinal‐color variation in this valley is two confounding factors, the accidental movement of individuals during, for example, construction, alongside changing local habitat, itself a consequence of the development of the valley. In the future, it should be possible to investigate further, for example by comparing the population genomic structure of the different valleys, and determine the putative role of selection in maintaining altitudinal clines, and the extent and contribution of random drift/founder effect in first establishing patterns, and the extent to which humans have intervened. These same studies could also be used to further understand the dynamics of the clines; previous studies using marked snails and models of the effect of drift and migration suggest that, irrespective of whether the cline is a due to altitudinal selection or of the meeting of separate founding populations, the clines themselves are rather old (Cameron et al., 2013).

The only other location that showed change was in the Ribagorzana valley, where the proportion of banded shells has risen from ~ 3% to 14%, with substantially fewer brown shells recorded and more yellow shells. The explanation for changes in this valley is not clear. One possibility is that we were more likely to score an intermediate shell as pink rather than brown compared with previous workers. However, this can probably be discounted because the lower proportion of recorded brown shells in our samples from Ribagorzana is matched by an increased proportion of yellow rather than pink shells. The general finding of reduced browns is perhaps in line with other studies. Cowie and Jones (1998) and Cook et al. (1999) documented an overall decrease in the frequency of the brown shells, Ożgo and Schilthuizen (2012) identified that brown shells decreased in expenses of yellow shells, Cameron et al. (2013) reported a general increase of yellows and Cook (2014) found an increase of yellows in woodland habitats.

### 4.3 From phenotype to genotype

One limitation of comparative studies on *Cepaea* is that there is a risk that we ascribe “just‐so” explanations to changes in the frequencies of a particular phenotype over time. For example, in this study, we could have concluded that the changes that we observed in Vielha valley may be due to immigration of new individuals (because of construction), but of course altered natural selection is also likely, especially because of changed habitat associated with the construction industry.

Recent progress in genomic technologies will certainly offer a solution, including the availability of a first draft *C. nemoralis* genome (Saenko et al., 2020). For example, it should be possible to use population genomics to understand the relative roles of migration/founder effect and selection in determining the population structure of *Cepaea* populations. In particular, these methods may be used to understand the history of a population; for example, Is there evidence for recent immigration to the high altitude regions of the Vielha valley, from snails that perhaps originate from elsewhere? Alternatively, is there evidence for a selective sweep at the loci that control the shell phenotype, perhaps indicative of a local response to a change in the selective regime?

Some of the other remaining issues that we have only touched upon here are the correlations between altitude and multiple phenotypic traits (banding, color, lip color, size, shape), as well as both linkage and linkage disequilibrium between the genes involved (Cook, 2013; Gonzalez et al., 2019). Given that lip color is ordinarily a dark color in *C. nemoralis* across most of Europe (with some exceptions), and that this is the main character that distinguishes this species from *C. hortensis*, the wide variation in this character in the Pyrenees is particularly mysterious. In the future, we hope to understand the genetic basis for these characters; it is hoped that this will bring forth an era in which we are better able to understand the impact of the multiple factors (Jones et al., 1977), including natural selection and random genetic drift, that determine the patterns of shell types that are present in nature.

## CONFLICT OF INTEREST

The authors have no conflict of interest to declare.

## AUTHOR CONTRIBUTION

**Daniel Ramos Gonzalez:** Conceptualization (equal); Data curation (equal); Formal analysis (equal); Investigation (equal); Methodology (equal); Writing‐original draft (equal); Writing‐review & editing (equal). **Angus Davison:** Conceptualization (equal); Data curation (equal); Formal analysis (equal); Funding acquisition (equal); Investigation (equal); Methodology (equal); Project administration (equal); Writing‐original draft (equal); Writing‐review & editing (equal).

## Supporting information

Table S1‐S4

## Data Availability

All raw data for this manuscript are supplied in the Supplementary Information, Tables S1–S4.
